# Association of thiol/disulphide homeostasis with Bethesda classification of thyroid nodules and thyroid cancer

**DOI:** 10.55730/1300-0144.5400

**Published:** 2022-03-19

**Authors:** Muhammet Cüneyt BİLGİNER, Abbas Ali TAM, Sevgül FAKI, Bağdagül Yüksel GÜLER, Özcan EREL, İbrahim KILINÇ, Didem ÖZDEMİR, Oya TOPALOĞLU, Reyhan ERSOY, Bekir ÇAKIR

**Affiliations:** 1 Department of Endocrinology and Metabolism, Faculty of Medicine, Karadeniz Technical University, Trabzon, Turkey; 2 Department of Endocrinology and Metabolism, Faculty of Medicine, Ankara Yıldırım Beyazıt University, Ankara, Turkey; 3 Department of Endocrinology and Metabolism, Ankara City Hospital, University of Health Sciences, Ankara, Turkey; 4 Department of Endocrinology and Metabolism, Gülhane Training and Research Hospital, University of Health Sciences, Ankara, Turkey; 5 Department of Medical Biochemistry, Faculty of Medicine, Ankara Yıldırım Beyazıt University, Ankara, Turkey; 6 Department of General Surgery, Ankara City Hospital, University of Health Sciences Turkey, Ankara, Turkey

**Keywords:** Thyroid cancer, oxidative stress, Bethesda, thiol/disulphide

## Abstract

**Background/aim:**

Ultrasonography and fine-needle aspiration biopsy are frequently used to diagnose thyroid cancer. However, supportive data might be required in case of diagnostic difficulty. This study investigated whether there is a relationship between thiol/disulphide homeostasis and cytological and histopathological diagnosis of thyroid nodules.

**Materials and methods:**

The patient group consisted of 81 individuals with euthyroid nodular (single/multiple) goiter scheduled for thyroidectomy, and the control group consisted of 28 age- and sex-matched healthy volunteers who had no thyroid nodule on ultrasonographic evaluation. All participants were selected among the admissions to the study clinic between June 2017 and June 2018, and venous blood samples were collected. The samples of the patients were taken before surgery. Thiol and disulphide levels were analysed with the automated spectrophotometric method.

**Results:**

The mean age of the patient group was 45.66 ± 10.45 years, and the mean age of the control group was 43.53 ± 11.49 years (p = 0.365). The increasing Bethesda categories were positively correlated with the disulphide level (r = 0.281, p = 0.011), disulphide/native thiol ratio (r = 0.241, p = 0.030) and disulphide/total thiol ratio (r = 0.250, p = 0.024). Disulphide/native thiol ratio and disulphide/total thiol ratio were significantly higher in the histopathologically malignant (euthyroid nodular goiter but final pathology reported malignant) compared to histopathologically benign (euthyroid nodular goiter but final pathology reported benign) (p = 0.012; p = 0.007, respectively) and control groups (p = 0.006; p = 0.004, respectively), but no significant difference was found in these ratios between benign and control group (p = 0.711; p = 0.749, respectively).

**Conclusion:**

Oxidative stress parameters were significantly higher in thyroid cancer. A positive correlation was detected between Bethesda categories with increased risk of malignancy and the disulphide/native thiol ratio and the disulphide/total thiol ratio.

## 1. Introduction

The frequency of detection of thyroid nodules is gradually increasing with the widespread use of ultrasonography [1,2]. Thyroid nodule size and malignancy risk are important parameters that should be evaluated during the management of thyroid nodules in euthyroid patients [3]. The gold standard method for the differentiation of benign and malignant nodules is fine-needle aspiration biopsy (FNAB) [3]. The cytological specimen obtained by biopsy is generally classified according to the Bethesda classification system [4]. Accordingly, there are six groups; nondiagnostic, benign, atypia of undetermined significance/follicular lesion of undetermined significance (AUS/FLUS), follicular neoplasia/suspicious for follicular neoplasia (FN/SFN), suspicious for malignancy (SM), and malignant [5]. The cytology result obtained upon this classification is combined with ultrasonography data, and a follow-up plan is developed. Particularly in nodules with indeterminate results, molecular markers such as B-Raf proto-oncogene mutation analysis may also be helpful in deciding the management [3]. However, many clinicians need further supportive data to manage euthyroid multinodular goiter. Thiols constitute a large part of antioxidants and provide defence against reactive oxygen species (ROS) through their role in redox homeostasis [6]. Thiols have the sulfhydryl group and may enter the oxidation reaction to form disulphide bonds [6]. Dynamic thiol-disulphide homeostasis reverses thiol oxidation in proteins and represents thiol and disulphide levels [6]. Dynamic thiol-disulphide plays a crucial role in many events such as homeostasis, antioxidant protection, detoxification, signal transduction, apoptosis, and regulation of enzymatic activity [6,7]. There is evidence that abnormal thiol-disulphide homeostasis plays a role in the pathogenesis of diabetes, cancer, rheumatic diseases, chronic kidney disease, and neurodegenerative diseases [8–13]. Therefore, determining dynamic thiol-disulphide homeostasis may provide information about various biochemical processes [6]. Only a small part of the total thiol in the organism consists of low molecular weight thiols. In plasma, total thiols are lower than in cells, and the predominant thiol is human serum albumin [14]. In 2014, Erel and Neşelioğlu developed a new automated spectrophotometric method in which two elements can be measured separately, thus representing both sides of the balance simultaneously [6]. Using this method, we aimed to determine whether there is a relationship between cytological findings and oxidative stress, and we investigated the role of oxidative stress in the differentiation of benign and malignant thyroid nodules.

## 2. Materials and methods

### 2.1. Participants

This study included 81 patients diagnosed with euthyroid nodular (single/multiple) goiter scheduled for thyroidectomy. These patients were selected after the thyroid surgery multidisciplinary council, in which an average of 30 thyroid patients were evaluated every week. The process was completed between June 2017 and June 2018. Thyroidectomy indications were the coexistence of giant nodule, malignant, SM, FN/SFN, AUS/FLUS, or persistent nondiagnostic cytology, and patients’ preference. Patients with hypothyroidism, hyperthyroidism, diabetes mellitus, cardiovascular diseases, cerebrovascular diseases, chronic kidney diseases, chronic liver diseases, rheumatic diseases, and malignancy were excluded from the study [15]. Patients on lipid-lowering drugs, patients who use alcohol, and smokers were excluded as well. Twenty-eight age- and sex-matched healthy volunteers who had no thyroid nodule on ultrasonographic evaluation formed the control group. Exclusion criteria were also applied to this group. Twelve-hour fasting venous blood samples of all the participants who met the inclusion criteria were collected between the study dates. The samples of the patient group were taken immediately after the council, before thyroidectomy. Preoperative thyroid functions [serum thyrotropin (TSH), free triiodothyronine (fT3), free thyroxine (fT4)], antithyroid peroxidase antibody (anti-TPOAb), antithyroglobulin antibody (anti-TGAb), cytology results, and histopathology results (malignant/benign) were obtained from medical records. Serum TSH, fT3, and fT4 levels were measured using chemiluminescence methods (Immulite 2000, Diagnostic Products Corp., Los Angeles, California and UniCel DXI 800, Beckman Coulter, Brea, California). Normal ranges for TSH, fT3, and fT4 were 0.27–4.2 uIU/mL, 1.57–4.71 pg/mL and 0.61–1.12 ng/dL, respectively. The study was conducted following the 2013 Brazilian version of the Helsinki Declaration and was approved by the Local Ethics Research Committee.

### 2.2. Thiol/disulphide homeostasis

Venous blood samples from the patients and healthy controls were collected after 12 h of fasting and put in EDTA tubes. Plasma samples were separated from cells by centrifugation at 1500 × *g* for 10 min. Samples were run immediately or stored at −80 °C. During the thiol/disulphide homeostasis tests, the reducible disulphide bonds were first reduced to form free functional thiol groups. Formaldehyde was used to remove unused and consumed sodium borohydride. Total thiol (−SH+−S−S) and native thiol (−SH) levels in the samples were measured using Ellman’s and modified Ellman’s reagent [16]. The natural thiol content was subtracted from the total thiol content, and half of this difference gave the amount of dynamic disulphide bonds (−S – S−). Using these parameters, disulphide/native thiol ratios (−S – S−) × 100/(− SH), disulphide/total thiol ratios (−S – S−) × 100/(− SH+−S – S−) and native thiol/total thiol ratios (−SH) × 100/(− SH+− S – S−) ratios were calculated.

### 2.3. Ultrasonography

Experienced endocrinologists performed ultrasonography (DO, RE) using Esaote color Doppler ultrasonography device (Model 796 FDII; MAG Technology Co. Ltd., Yung-Ho City, Taipei, Taiwan) and a superficial probe (Model LA523 13-4 5.5–12.5 Mhz).

### 2.4. Fine-needle aspiration biopsy and cytology

FNAB was performed under ultrasonography (Logic Pro 200 GE and 7.5 MHz probes; Kyunggigo, Korea) guidance with a 27-gauge needle and a 20-mL syringe. Samples were air-dried and stained with Giemsa stain. Bethesda classification system was used for cytological diagnosis [4]. In patients with multiple nodules, the nodule carrying the highest risk of malignancy according to the Bethesda classification was taken into consideration. In this study, malignancy risk was coded as ordinal data from 1 to 6 (1 = benign, 2 = nondiagnostic, 3 = AUS/FLUS, 4 = FN/SFN, 5 = SM, 6 = malignant) according to cytology results. Malignancy risk was accepted to increase as the Bethesda category went from 1 to 6.

### 2.5. Histopathology

The histopathological evaluation was made according to the 2004 World Health Organization criteria [17].

### 2.6. Statistical analysis

All statistical analyses were performed with the SPSS 15.0 software package (SPSS, Inc., Chicago, Illinois). Descriptive statistics for the continuous variables were expressed as mean ± SD or median (range), and categorical variables were noted as numerics and percentages (%). Student’s t-test was used to compare independent groups with normal distribution, and the Mann–Whitney U test was used for those that are not normally distributed. Chi-square was used to compare categorical variables. The Kruskal–Wallis test was used when comparing three or more independent groups without normally distributed data, and the one-way ANOVA test was used for normally distributed data. Post hoc Dunn’s test with Bonferroni correction was performed. p < 0.05 was considered to be statistically significant. Spearman correlation test was used to present the relationship between malignancy risk and thiol/disulphide homeostasis.

## 3. Results

There were 57 women (70.4%) and 24 men (29.6%) in the patient group (n = 81), and 19 women (67.9%) and 9 men (32.1%) in the control group (p = 0.803). The mean age of the patient group was 45.66 ± 10.45 (18–65) years, and the mean age of the control group was 43.53 ± 11.49 (24–64) years (p = 0.365). There was no difference between the groups regarding body mass index, TSH, fT3, fT4 (Table-1). Anti-TG was positive in 19.8% of the patients, and anti-TPO was positive in 16% of the patients; however, there was no significant difference between positive autoantibody patients vs. whole negative population for thiol/disulphide ratio (p > 0.05). According to the ultrasonography findings, solitary nodules were detected in 15 (18.5%) patients, while multinodular goiter was detected in 66 (81.5%) patients. Cytological diagnosis was nondiagnostic (recurrent) in 23 (28.4%), benign in 17 (21.0%), AUS/FLUS (recurrent) in 20 (24.7%), FN/SFN in 5 (6.2%), SM in 8 (9.9%), and malignant in 8 (9.9%) patients. Native thiol, total thiol, and disulphide levels, disulphide/native thiol, disulphide/total thiol, and native thiol/total thiol ratios according to cytological results are given in Table 2. There was no significant difference in terms of these parameters between cytology groups. Significant positive correlations were detected between increasing risk of malignancy according to cytology and disulphide (r = 0.281, p = 0.011), disulphide/native thiol (r = 0.241, p = 0.030) and disulphide/total thiol (r = 0.250 p = 0.024) (Table 3). On the other hand, there was no significant correlation between age, BMI, albumin level of patients, and their oxidative stress parameters (p > 0.05). Thyroidectomy was performed in 69 (85.2%) out of 81 patients. Histopathological diagnosis was benign in 34 (49.3%) and malignant in 35 (50.7%) patients. There was papillary thyroid cancer in 32 (91.5%) patients and tumour with uncertain malignant potential in 3 (8.5%) patients. The mean age was 45.8 ± 11.0 years the malignant patients and 46.3 ± 11.0 years in the benign group. In addition, 65.7% (n = 23) of the patients in the malignant group and 73.5% (n = 25) of the patients in the benign group were female. There was no statistical difference between the benign and malignant groups according to age and sex (p = 0.852; p = 0.481, respectively). Thiol and disulphide parameters in histopathologically benign and malignant patients and the control group are shown in Table 4. There were statistically significant differences in the disulphide/native thiol ratio and disulphide/total thiol ratio between groups (p < 0.001 for all). Disulphide/total thiol and disulphide/native thiol were significantly higher in the malignant compared to the benign (p = 0.012; p = 0.007, respectively) and the control groups (p = 0.006; p = 0.004, respectively), but no significant difference was found between the benign and the control groups (p = 0.711; p = 0.749, respectively).

## 4. Discussion

To our knowledge, this study is the first to compare thiol/disulphide homeostasis in patients with euthyroid nodular goiter and in healthy subjects. An increase in free radicals is associated with alteration in protein function, lipid denaturation, and structural damage to DNA. These changes increase the risk of mutation and neoplastic transformation [18,19]. B-Raf proto-oncogene - Ras proto-oncogene point mutations and rearranged during transfection (RET) gene fusions, which are the most frequently activated oncogenes in papillary thyroid cancer (PTC), activate the mitogen-activated protein kinase (MAPK) signalling pathway. Oxidative stress is considered a starting point for deoxyribonucleic acid (DNA) damage which is the first step for tumorigenesis [20]. It was shown that serum ischemia with modified albumin (IMA) levels was increased in both hypothyroid and hyperthyroid patients compared to euthyroid controls in a metaanalysis [21]. Therefore, we excluded patients with thyroid dysfunction (subclinical and overt) and only included euthyroid patients in our study. A study conducted in Turkey found that serum malondialdehyde (MDA) level, which is a marker for lipid peroxidation, was significantly higher in patients with euthyroid multinodular goiter compared to the control group, and it decreased after thyroidectomy [22]. In addition, it was shown that MDA level is significantly higher in papillary carcinoma samples than in normal thyroid tissue [23]. In our study, preoperative disulphide/native thiol ratio and disulphide/total thiol ratio were significantly higher in patients with malignant histopathology compared to the patients with benign histopathology and control group, while no significant differences were observed between the benign group and the control group. Wang et al. reported that oxidative stress index was a determining risk factor in patients with thyroid cancer than benign nodular goiter and healthy control group [24]. In another study, significantly higher free radicals were shown in tumour thyroid tissue than the healthy thyroid tissue [25]. In a study conducted in Turkey evaluating thiol/disulphide homeostasis; native thiol and total thiol were lower, while disulphide was higher in patients with thyroid cancer than in the control group (n = 23) [26]. However, statistical data were not provided in that study, probably because the number of patients was not sufficient for statistical analysis. Again, in a study from Turkey, superoxide dismutase (SOD) was lower in various thyroid disorders, including thyroid cancer [27]. The relevant literature, including our study, suggests that oxidative stress markers analysed with different methods are increased in patients with thyroid cancer. In the study by Erel and Neselioğlu, the most striking feature of thiol/disulphide balance is that the analysis was performed by removing sodium borohydride using formaldehyde [6]. We believe that we reflected the thiol/disulphide homeostatic state better than previous studies since the two elements could have been measured separately with our method.

It has been suggested that the causes of the increase in oxidative stress in thyroid cancer are the increased lipid peroxidation and damage to the antioxidant defence system [28]. Nicotinamide adenine dinucleotide phosphate (NADPH) oxidases (Dual oxidase 1, Dual oxidase 2, NADPH oxidase 4) are expressed in the human thyroid gland and function as sources of ROS, suggesting that they may play a role in the pathogenesis of thyroid diseases [29]. The direct interaction of ROS with DNA may lead to oxidative DNA damage, including abasic regions and single-stranded DNA breaks [30]. ROS sources in thyroid tumour formation have been identified as dual oxidase 1 and NADPH oxidase 4. The dysregulation of their expression and activity may jeopardize DNA stability and affect cell fate [29]. Chemical modifications of the DNA structure, such as the presence of 8-oxo-2′-deoxyguanosine as induced by oxidative stress, have been found specifically in thyroid cancer [31].

According to the Bethesda scoring, the thiol/disulphide homeostasis has not been previously evaluated. Our study is the first attempt in that regard. Malignancy risks in different Bethesda categories are predicted as follows; nondiagnostic <1%–4%, benign <1%, AUS/FLUS 5%–10%, FN/SFN 20%–30%, SM 60%–75%, and malignant cytology 97%–99% [3]. In our study, no significant difference was found between Bethesda categories in terms of thiol-disulphide homeostasis. However, disulphide/native thiol ratio (r = 0.241, p = 0.030) and the disulphide/total thiol ratio (r = 0.250, p = 0.024) were positively correlated with Bethesda categories with increasing malignancy risk. We think that oxidative stress marker analysis in Bethesda subgroups can be employed in a further study with a larger patient population to enlighten this issue. Clarification of this situation in the euthyroid multinodular patient group may benefit many cases.

In conclusion, preoperative disulphide/native thiol and disulphide/total thiol markers reflecting the oxidative damage were significantly higher in the malignant group compared to the benign and control groups. Bethesda classification with increasing risk of malignancy was positively correlated with disulphide/native thiol and the disulphide/total thiol.

## 5. Limitations

The main limitation of this study is its single-centred design which makes it difficult to generalize the study results. Secondly, no comparison was made with other oxidative stress markers such as MDA and SOD because they were not included in the analysis. Thirdly, there were relatively few patients in the Bethesda subgroups, which might have caused no difference in oxidative stress between the Bethesda categories. Examining the relationship between Bethesda categories and thiol/disulphide homeostasis in a single Bethesda group with a larger patient population may be more informative.

## Figures and Tables

**Table 1:** Demographical features and laboratory findings of the study population.

Variables	Euthyroid nodular goiter (n=81)	Control (n = 28)	p-value

Sex, n (%)			
female	57 (70.4%)	19 (67.9%)	0.803
male	24 (29.6%)	9 (32.1%)	

Age (years)	45.66 ± 10.45	43.53 ± 11.49	0.365

BMI (kg/m^2^)	28.49 ± 3.95	26.96 ± 4.78	0.099

TSH (μIU/mL)	1.72 ± 0.87	1.78 ± 0.58	0.706

fT4 (ng/dL)	1.28 ± 0.17	1.23 ± 0.18	0.295

fT3 (ng/dL)	3.26 ± 0.46	3.37 ± 0.35	0.249

Anti-TG positivity	16 (19.8%)	0 (%)	-

Anti-TPO positivity	13 (16.0%)	0 (%)	-

Total Bilirubin (mg/dL)	0.60 ± 0.49	0.58 ± 0.29	0.298

Albumin (g/dL)	4.61 ± 0.30	4.80 ± 0.35	0.001

CRP (mg/L)	1.48 ± 2.56	1.39 ± 2.31	0.965

BMI: Body mass index, TSH: Serum thyrotropin, fT4: Free thyroxine, fT3: Free triiodothyronine, Anti-TG: Anti-thyroglobulin antibody, AntiTPO: Anti-thyroid peroxidase antibody, CRP: C-reactive protein.

**Table 2:** Thiol/disulphide homeostasis in different Bethesda categories.

Variables	Non diagnostic (n = 23)	Benign (n = 17)	AUS/FLUS (n = 20)	FN/SFN (n = 5)	SM (n = 8)	Malign (n = 8)	p value
Native Thiol (μmol/L)	461.25 **±** 57.61	453.57 ±64.87	470.20±63.57	415.40**±** 53.18	435.02**±**69.33	425.35**±**56.65	0.322
Total thiol (μmol/L)	494.40 ± 59.20	490.72 ± 76.19	504.31 ± 63.09	472.76 ± 52.59	472.22 ± 79.16	459.6 ± 57.67	0.552
Disulphide (μmol/L)	17.94 ± 9.69	16.49 ± 7.50	17.55 ± 7.14	24.68 ± 8.18	24.59 ± 7.7	25.37 ± 9.07	0.655
Disulphide/ Native thiol (%)	3.97 ± 2.28	3.75 ± 1.81	3.83 ± 1.65	6.39 ± 5.71	5.81 ± 2.20	6.15 ± 2.64	0.251
Disulphide/ Total thiol (%)	3.62 ± 1.90	3.43 ± 1.60	3.52 ± 1.43	5.28 ± 4.14	5.37 ± 2.11	5.60 ± 2.14	0.253
Native thiol/ Total thiol (%)	93.30 ± 4.05	92.58 ± 3.46	93.17 ± 3.09	87.97 ± 7.87	92.25 ± 2.77	92.62 ± 5.30	0.405

AUS/FLUS: Atypia of undetermined significance/follicular lesion of undetermined significance, FN/SFN: Follicular neoplasia / suspicious for follicular neoplasia, SM: Suspicious for malignancy.

**Table 3:** Correlation between Bethesda categories with increasing risk of malignancy and thiol/disulphide homeostasis.

Variables	Disulphide	Native thiol	Total thiol	Disulphide/ native thiol	Disulphide/ Total thiol	Native thiol/ total thiol

Bethesda						
r	0.281	−0.122	−0.073	0.241	0.250	−0.002
p	0.011	0.276	0.516	0.030	0.024	0.986

**Table 4:** Comparison of thiol/disulphide homeostasis in histopathologically benign and malignant patients and control group.

Variables	Benign (n = 34)	Malignant (n = 35)	Control (n = 28)	p
Native Thiol (μmol/L)	453.19 ± 63.49	457.47 ± 62.38	444.81 ± 71.9	0.477
Total thiol (μmol/L)	487.45 ± 67.87	497.09 ± 64.78	474.61 ± 75.48	0.256
Disulphide (μmol/L)	16.07 ± 9.28	19.85 ± 11.28	14.87 ± 7.62	0.191
Disulphide/native thiol (%)	3.58 ± 2.07	5.50 ± 2.85	3.41 ± 1.70	0.002^a^
Disulphide/total thiol (%)	3.27 ± 1.80	4.96 ± 2.24	3.15 ± 1.48	0.001^b^
Native thiol/total thiol (%)	93.01 ± 3.96	92.07 ± 4.73	93.74 ± 3.09	0.473

a Significance between the malignant group and the benign group (p = 0.012) and the malignant group and the control group (p = 0.006) (pairwise comparison).

b Significance between the malignant group and the benign group (p = 0.007) and the malignant group and the control group (p = 0.004) (pairwise comparison).
